# Research progress on histone deacetylases in peritoneal dialysis-associated peritoneal fibrosis

**DOI:** 10.1080/0886022X.2025.2597681

**Published:** 2025-12-18

**Authors:** Wenhui Qiu, Jiayi Chen, Tapas Ranjan Behera, Ying Huang, Chenling Chu, Baihui Xu, Quanquan Shen, Jingwen Yan

**Affiliations:** a Basic Medical Sciences, Hangzhou Medical College, Hangzhou, Zhejiang, China; b Department of Clinical Medicine, Hangzhou Normal University, Hangzhou, Zhejiang, China; c Department of Cancer Biology, Cleveland Clinic, Cleveland, OH, USA; d Department of Public Health and Preventive Medicine, Hangzhou Medical College, Hangzhou, Zhejiang, China; e Urology & Nephrology Center, Department of Nephrology, Zhejiang Provincial People’s Hospital (Affiliated People’s Hospital, Hangzhou Medical College), Hangzhou, Zhejiang, China; f Department of Nephrology, Zhejiang Provincial People’s Hospital Bijie Hospital, Bijie, Guizhou, China

**Keywords:** Peritoneal dialysis, peritoneal fibrosis, epithelial-mesenchymal transition, epigenetics, histone deacetylases

## Abstract

When chronic kidney disease (CKD) progresses to end-stage renal disease (ESRD), peritoneal dialysis (PD) can serve as an effective alternative therapy, but it also has its limitations. Peritoneal fibrosis (PF), a PD-related complication, is one of the major and serious complications of long-term PD that can lead to ultrafiltration failure, severely impacting the efficacy of PD treatment. At present, most of the research on the molecular mechanisms of fibrosis focuses on the liver and kidney, but there is relatively little research on PF for identifying anti-fibrotic targets. Histone deacetylase (HDAC), as an enzyme that exerts transcriptional regulation through deacetylation, when activated can lead to the occurrence and development of inflammation and fibrosis. This review aims to assess the effects of HDAC and HDAC inhibitors on peritoneal inflammation and fibrosis in PF. Therefore, we reviewed the recent progress in PF treatment, focusing on understanding the characteristics and functions of HDAC and their interactions with the extracellular matrix in PF progression, and explored the crucial role of HDAC in regulating fibrosis regression. Additionally, we explored future research directions to identify potential methods for treating PF.

## HIGHLIGHTS

1. The process of peritoneal fibrosis involves multiple mechanisms.
2. A specific overview of the histone deacetylase family.
3. Different classes of histone deacetylases have varying mechanisms of action in peritoneal fibrosis.

## Introduction

In recent years, chronic kidney disease (CKD) has become an important public health problem, and progression of CKD leading to end-stage renal disease (ESRD) has a high mortality rate, and seriously endangers human life and health. As an effective renal replacement therapy for ESRD [1], peritoneal dialysis (PD) has been widely recognized by patients due to its significant advantages, such as ease of operation, home-based dialysis, and preservation of residual renal function [2]. However, clinical studies have shown that due to prolonged contact with biologically incompatible peritoneal dialysis fluid (PDF) and the influence of factors such as peritoneal inflammation and uremic toxins, the integrity of peritoneal function is compromised, and the structure and function of the peritoneum become disordered, thereby leading to the occurrence of peritoneal fibrosis (PF) [3,4]. The occurrence and development of PF is a long-term process: epithelial cells undergo epithelial-mesenchymal transition (EMT), fibroblasts are continuously activated, and extracellular matrix (ECM) is continuously deposited [5].

Epigenetic modifications have always played a crucial role in the occurrence and development of human diseases. In recent years, research on histone modifications has provided new ideas for disease treatment [6,7]. Among them, acetylation is the most important and widespread modification method of histones [8]. Histone deacetylase (HDAC) is an important enzyme in the process of histone modification. By removing the acetyl groups from the acetylated lysine residues in histones and non-histones, it plays a crucial role in regulating histone acetylation. When the level of histone deacetylation is elevated due to certain stimuli, the normal cell cycle and metabolic behaviors in the body will change, thereby inducing tumors and fibrotic lesions [9]. Studies have shown that HDAC is a key factor in regulating gene expression and cell proliferation during the inflammatory response, and is involved in the fibrotic process of organs (Table 1) [26]. HDAC inhibitors (HDACi) (such as vorinostat and romidepsin) have good therapeutic effects on inflammatory diseases, indicating that HDAC does indeed have therapeutic potential in treating organ fibrosis. However, there is currently a lack of specific inhibitors of the HDAC family, and some selective inhibitors also have differing opinions regarding subtype selection [12,27]. Recent studies have shown that during the disease progression of PF, compared to the normal state, the sites and levels of histone acetylation have significantly changed [28,29]. Similarly, histone acetyltransferase (HAT) and HDAC play important roles in regulating the proliferation and activation of peritoneal mesothelial cells [30,31]. These changes indicate that HDAC is involved in the interaction process of various cells in the fibrotic process of peritoneal tissue, confirming its importance in the pathogenesis of fibrosis.

**Table 1.** Preclinical effects of the HDACs superfamily in the regulation of PF.

Class	Damage/disease model	Targets	Mechanism	References
HDAC1	PMCs	MMT	HDAC1 promotes the upregulation of mesenchymal markers (MMP2, Col1A1, PAI-1, TGF-β1) and the downregulation of epithelial markers (E-cadherin, Occludin), thereby promoting MMT.	Ross et al. (2018) [10]
	PMCs, Met-5A	HDAC1-WT1- mir −769-5p	The expression of WT1 and its binding to the miR-769-5p promoter are both increased by HDAC1 inhibition, and the ectopic expression of miR-769-5p promotes the reversal of MMT.	Giulio et al. (2022) [11]
HDAC2	Chronic interstitial fibrosis in renal transplantation	TNF-α	The multimolecular complex formed by HDAC2 and YY1 can reverse the expression of EMT markers in a mouse chronic renal allograft immunofluorescence model.	Zhang et al. (2023) [12]
HDAC3	PMCs	Wnt/β-Catenin	1,25(OH)₂D₃ prevents EMT in PMCs by inhibiting HDAC3 and increasing vitamin D receptor (VDR) expression *via* the Wnt/β-Catenin signaling pathway.	Liu et al. (2019) [13]
	PMCs	Inflammatory factors	CHP can reduce markers of fibrosis and inflammation by regulating HDAC3 expression and related signaling pathways.	Kim et al. (2024) [14]
HDAC8	PF	EGFR	HDAC8 promotes phosphorylation of epidermal growth factor receptor (EGFR) and activation of its downstream signaling pathways ERK1/2 and STAT3/HIF-1α, increases apoptosis, and promotes EMT.	Zhou et al. (2023) [15]
HDAC4	UUO	Cell cycle	HDAC4 promotes renal tubular epithelial cell arrest and TGF-β1 expression in the G2/M phase of the cell cycle.	Shen et al. (2022) [16]
HDAC5	–	NF-κB	HDAC5 acts on the NF-κB pathway, enhancing the phosphorylation and degradation of IκB (an inhibitor of NF-κB) because the acetylation of IκB can inhibit its ubiquitination and degradation, thereby indirectly promoting the nuclear translocation of NF-κB and supporting the survival of PMCs.	Xu et al. (2021) [17]
HDAC7	Human lung fibroblast	PGC1a	HDAC7 deacetylates histone H3 near the promoter of the anti-fibrotic gene *PGC1α,* thereby inhibiting the expression of anti-fibrotic genes such as PGC1a, and ultimately promoting fibroblast activation and proliferation.	Jones et al. (2019) [18]
HDAC6	PMCs	NF-κB, TGF-β1/Smad	The overactivation of HDAC6 can limit the pro-fibrotic effects of TGF-β1 by inhibiting the nuclear transport of Smad in a compensatory manner; however, over the long term, it may exacerbate damage due to dysregulation in other pathways, such as NF-κB.	Xu et al. (2017) [19]
	PMCs	Wnt/β-Catenin	HDAC6 facilitates the deacetylation of α-tubulin, leading to a loose and unstable microtubule structure, which inhibits the efficient transport of β-catenin to the nucleus *via* microtubules, thereby suppressing pathway activity and the expression of downstream antifibrotic genes.	Shi et al. (2020) [20]
SIRT1	PF	TGF-β	Upregulation of SIRT1 can effectively ameliorate PF by inhibiting TGF-β signal-induced secretion of protein matrices.	Guo et al. (2021) [21]
	PMCs	AMPK/SIRT1	AMPK directly phosphorylates Smad3, inhibiting its binding to the TGF-β receptor; SIRT1 further diminishes its transcriptional activity through deacetylation of Smad3.	Lu et al. (2024) [22]
SIRT2	Pulmonary fibrosis	Smad2/3	SIRT2 can directly deacetylate Lys378 of Smad3 (a key site for acetylation), thereby reducing its binding affinity with Smad4 and diminishing the formation of the ‘Smad2/3-Smad4’ complex, which inhibits its transport into the nucleus and reduces the release of pro-inflammatory factors such as TNF-α and IL-6.	Gong et al. (2021) [32]
SIRT3	Myocardial fibrosis	TGF-β	The downregulation of SIRT3 weakened the anti-fibrotic ability and promoted the release of cytokines, which in turn aggravated the progression of myocardial fibrosis.	Ma et al. (2018) [33]
SIRT4	Myofibroblasts	Gln	SIRT4 regulates myofibroblast transdifferentiation and subsequent collagen fibrillary deposition by regulating glutamine (Gln) metabolism.	Luo et al. (2016) [23]
SIRT6	PMCs	MMT	SIRT6 expression can inhibit MMT in PMCs and alleviate PF.	Shi et al. (2024) [24]
HDAC11	Renal tubular epithelial cells	TGF-β	HDAC11 promotes the expression of pro-fibrotic genes.	Mao et al. (2020) [25]

HDAC, histone deacetylase; MMT, mesothelial-to-mesenchymal transition; MMP2, matrix metallopeptidase 2; Col1A1, Collagen I; TGF-β1, transforming growth factor beta receptor 1; TNF-α, tumor necrosis factor-α; EMT, epithelial-mesenchymal transition; PF, peritoneal fibrosis; EGFR, epidermal growth factor receptor; P65, RelA; HIF-1α, hypoxia inducible factor-1α; PMCs, peritoneal mesothelial cells; NF-κB, Nuclear factor kappa B; IκB, inhibitor of NF-κB; PD, peritoneal dialysis; TGF-β, transforming growth factor-β; AMPK, Adenosine 5′-monophosphate (AMP)-activated protein kinase; SIRT, Silent mating type information regulation 2 homolog; IL-6, interleukin-6; Smad, drosophila mothers against decapentaplegic protein.

At present, most studies still focus on the overall activity of HDAC, lacking specific mechanism analysis of the functional differences among different subtypes in the peritoneal microenvironment (high glucose and inflammatory factor enrichment). This article aims to fill this theoretical gap and address the current situation of “insufficient research on non-histone modifications in PF”. Therefore, developing specific, highly effective, and low-toxic subtype-selective HDACi has become a research hotspot in recent years and is urgently needed for application in the treatment of tumors and fibrotic diseases. We conducted a search of PubMed, Embase, and Web of Science to build our own database up to July 2025. The search terms included: (“Peritoneal fibrosis” OR “HDAC” OR “Epithelial-mesenchymal transition”) AND (“Peritoneal dialysis”), with no language restrictions. The initial search yielded 545 articles, and after removing duplicates, 370 remained. We then screened titles and abstracts, excluding 170 articles. The remaining 200 articles underwent full-text evaluation, with inclusion criteria being studies on PD-related PF patients, use of HDACi and publication between 2010 and 2025. Ultimately, 86 studies were included. In this review, we discuss the mechanism of action of HDAC and HDACi, the occurrence mechanism of PF, the potential mechanisms and functional differences of different HDAC subtypes and their inhibitors in PF, and discuss the key role of HDAC in mediating fibrosis regression, as well as the future research approaches of highly selective HDACi, in order to clarify the potential methods for precise targeting of PF treatment. This will provide new ideas for the treatment of PF.

## Overview of histone acetylation

Histones are a group of proteins rich in lysine and arginine residues in the nucleus, and post-translational modifications such as acetylation, methylation, and phosphorylation occur frequently. As the basic structural protein of chromosomes, they are mainly divided into five types in eukaryotic cells, namely H1, H2A, H2B, H3 and H4, all of which have their own acetylation sites: H2B has the highest acetylation level, with a total of 8 acetylation sites; H2A, H3, and H4 have 5, 3, and 4 acetylation sites, respectively [34]. Histones in eukaryotes work together with DNA to form nucleosome structures and chromatin. Chromatin can be further divided into euchromatin and heterochromatin. Euchromatin has a loose structure with active gene expression while heterochromatin is dense and unfavorable for gene expression. Therefore, some studies have shown that the structural density of chromatin is partly dependent on epigenetic modifications of histones [35]. Thus, to some extent, histone acetylation plays a key role in transcriptional regulation by regulating the degree of chromatin condensation.

Numerous studies have shown that among the numerous post-translational modifications of histones, histone acetylation is a key regulatory node for the core pathological features of PF [36–38]. HAT and HDAC dynamically regulate the level of histone acetylation. HDAC removes the acetyl groups from ε-n-acetyl-lysine residues on histones, increasing the positive charge on the residues. Since DNA molecules carry a negative charge, this enhances the binding affinity between histones and DNA molecules, thereby regulating histone acetylation levels and modulating downstream gene transcription, such as the *CHD1*, *COL1A1*, *IL6* and *TNF-α*. HAT, in contrast, works in the opposite manner [39]. In addition, HDAC not only acts on histones but can also deacetylate many non-histone proteins, such as tubulin and some transcription factors (such as p53), thereby regulating gene expression, altering cellular signal transduction, and controlling a series of biological processes including disease progression [40]. HDACi can regulate the expression and stability of proteins related to apoptosis and differentiation by increasing histone acetylation in specific chromatin regions, inducing apoptosis and differentiation [41], and have become a new research hotspot in the targeted treatment of inflammatory diseases.

It is worth noting that the HDAC family is mainly composed of 18 members from the HDAC and Sirtuin families. Based on their enzyme activity, sequence homology, and cellular localization, the 18 enzymes of the HDAC family can be classified into four classes: I, II, III, and IV. Class I includes HDAC1, 2, 3, and 8, which are mainly located in the cell nucleus [42]. Their catalytic domains are located between the short N-terminal and C-terminal domains, and they have a high degree of homology with the yeast RPD3-like enzymes. Class II can be further divided into Class IIa and Class IIb based on their catalytic regions. Class IIa (HDAC4, 5, 7, 9) has one catalytic region and is primarily located in the cell nucleus. They are generally in a non-phosphorylated state and become phosphorylated in response to specific signals. Class IIb (HDAC6, 10) has two catalytic regions and participates in the regulation of microtubule and actin-dependent cell movement. They shuttle between the nucleus and the cytoplasm and have homology with the yeast HDA-1-like enzymes [43]. Class III HDAC, also known as Sirtuins (SIRTs), contains seven isoforms, including Sirtuins 1–7, and are highly homologous to the yeast SIR2 and are NAD^+^-dependent HDACs. Class IV HDAC contains only one isoform, HDAC11. Class IV (HDAC11) has the same conserved sequence as Class I and Class II, but has higher tissue specificity and is highly expressed in the brain, heart, testis, and kidneys. Class I, II, and IV are Zn^2+^-dependent HDACs and have similar deacetylase domains, indicating that a single compound can simultaneously inhibit Zn^2+^-dependent HDACs, while Class III HDACs depend on nicotinamide adenine dinucleotide (NAD^+^) during the catalytic process [44,45].

Currently, histone deacetylation modification is gradually becoming a major research hotspot in disease treatment. This provides promising development prospects for treating PF by regulating HDACs.

## Mechanism of PF

**Figure 1.**
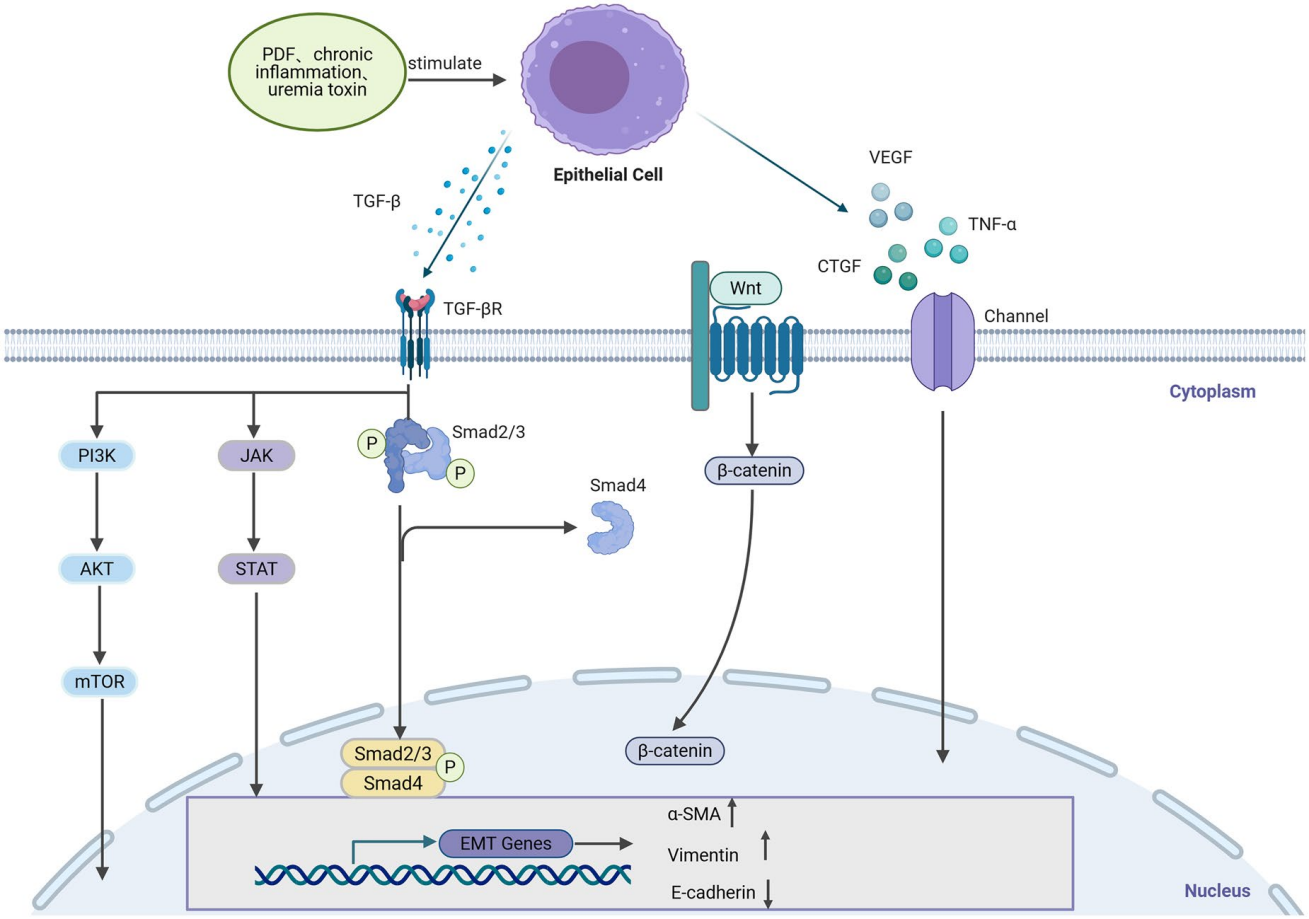
The signaling pathways involved in the process of fibrosis. PMCs secrete a large number of cytokines, such as TGF-β, VEGF, connective tissue growth factor (CTGF), and TNF-α, under stimulation by factors like PDF, peritoneal inflammation, and uremic toxins, each acting on different signaling pathways. In the typical TGF-β/Smad signaling pathway, TGF-β binds to TGFR2 after its release, which subsequently recruits and activates TGFR1. Active TGFR1 phosphorylates Smad2 and Smad3, forming a complex with Smad4 that translocates to the nucleus. The Smad3 component within the complex directly binds to gene promoters, inducing the production of molecules such as α-SMA. HDAC inhibitors can indirectly inhibit the phosphorylation of SMAD2/3 by maintaining the acetylation of Smad3, thereby enhancing the regulation of target genes (such as fibrosis-related gene *COL1A1* and *α-SMA*) by the TGF-β/Smad pathway. In non-Smad signaling pathways, activation of the PI3K-AKT-mTOR and JAK-STAT pathways can impact the coding of genes for pro-fibrotic factors. HDAC1/2 can deacetylate FOXO (a transcription factor that promotes the expression of apoptotic genes), leading to its phosphorylation (activation) by AKT, resulting in nuclear export and degradation, which inhibits PMCs apoptosis; HDAC can indirectly enhance the phosphorylation of STAT3 by JAK by inhibiting the expression of ‘negative regulatory factors of JAK kinases’ (such as SOCS3) through histone deacetylation that compresses the SOCS3 promoter, causing sustained activation of STAT3, which then enters the nucleus to initiate the expression of target genes (such as *α-SMA*). The Wnt/β-catenin signaling pathway promotes the accumulation of β-catenin in the cytoplasm. HDAC, histone deacetylase; PMCs, peritoneal mesothelial cells; PDF, peritoneal dialysate fluid; VEGF, vascular endothelial growth factor; TGF-β, transforming growth factor-β; TNF-α, tumor necrosis factor-α; CTGF, connective tissue growth factor; PI3K, Phosphoinositide 3-Kinase; AKT, Protein Kinase B; mTOR, mammalian target of rapamycin; JAK, Janus kinase; STAT, Signal transducer and activator of transcription, Smad2, drosophila mothers against decapentaplegic protein2; Smad3, drosophila mothers against decapentaplegic protein3; Smad4, drosophila mothers against decapentaplegic protein4; EMT, epithelial–mesenchymal transition; α-SMA, α-Smooth muscle actin.

In the course of long-term PD treatment, the occurrence of PF is the final result of a combination of factors. Due to the long-term bioincompatibility of PDF (high glucose, glucose degradation products, lactate buffer, acidic pH, and hypertonicity), peritoneal inflammation, uremic toxins and other factors, these stimulate cause PMCs to undergo EMT, increase the number of fibroblasts and produce a large amount of ECM, eventually leading to PF [46]. When PMCs are stimulated, they secrete numerous cytokines such as transforming growth factor-β (TGF-β) and vascular endothelial growth factor (VEGF), which further promote fibrosis of peritoneal tissue [47]. TGF-β plays a key role in the regulation of EMT [48], and VEGF is involved in the angiogenesis associated with PD [49]. HDACs have been shown to trigger the secretion of profibrotic cytokines, such as interleukin (IL)-1β, IL-6 and tumor necrosis factor-α (TNF-α), which are key regulators of inflammation and fibrosis [50,51], but the specific role of HDACs in the pathogenesis of PF remains uncertain (Figure 1).

### Peritoneal inflammation

PD-associated peritonitis is the most prevalent and serious complication of PD and a direct or major cause of death in approximately 16% of patients with PD [52]. PF can occur in patients treated with long-term PD due to acute peritonitis resulting from bacterial, fungal, or viral infections or improper procedures, as well as peritoneal microinflammation caused by prolonged exposure to bioincompatible PDF. Importantly, peritoneal inflammation occurs repeatedly, extensively damaging PMCs and causing PF. Preclinical research evidence indicates that Yu et al. [53] established a mouse model of PD-related peritoneal injury by intraperitoneal injection of human peritonitis PD effluent into male C57BL/6 mice daily for 6 weeks, and found that the peritoneal thickness of mice in the peritonitis effluent group was significantly greater than that in the control group. Immunohistochemical staining showed that the expression of PF markers (α-smooth muscle actin (α-SMA), Collagen I), neutrophils (MPO) and macrophages (CD68, F4/80) in the peritonitis effluent group was increased, and real-time qPCR showed significantly elevated expression of inflammatory markers (IL-1β, IL-6) and fibrosis markers (TGF-β1, α-SMA). More importantly, the amount of peritoneal ultrafiltration in the peritonitis effluent group was significantly reduced, suggesting that peritonitis can cause PF and affect peritoneal ultrafiltration capacity. It is highly likely that the high concentrations of glucose in the PDF and factors such as uremic toxins during long-term PD treatment contribute to a state of chronic inflammation in PD patients. Interestingly, research by Fang et al. [54] has demonstrated that PDF can significantly increase the infiltration of inflammatory cells (macrophages and neutrophils) in a PD rat model, as well as enhance the expression levels of pro-inflammatory cytokines (IL-6 and TNF-α) and chemokines. Furthermore, after being activated by lipopolysaccharide (LPS), peritoneal macrophages cause HDACs to rapidly bind to p65, enhancing the transcriptional activity of nuclear factor kappa B (NF-κB), which leads to an ‘explosive’ release of pro-inflammatory factors and exacerbates peritoneal congestion and exudation [55]. This suggests that in PD treatment, the activation of macrophages and the release of inflammatory cytokines can be stimulated to initiate an inflammatory response, although the specific role of HDACs in this process has not been clearly elucidated. Furthermore, chronic inflammation is a driving force behind the loss of PMCs, vascular lesions, and fibrotic changes in the peritoneum [56].

### Neovascularization

Peritoneal neovascularization is a common structural change in PF, wherein VEGF, as an effective promoter of endothelial cell growth, can stimulate the formation of new capillaries, leading to peritoneal angiogenesis and PF, and serves as the primary mediator for peritoneal neovascularization [57]. During the course of clinical treatment, patients undergoing PD treatment are highly likely to experience stimulation of PMCs, which secrete large amounts of VEGF due to prolonged exposure to bioincompatible PDFs, thereby affecting peritoneal blood vessels, leading to vasodilation, increased permeability, and a reduction in peritoneal ultrafiltration capacity. Research conducted by Li et al. [58] has demonstrated that patients undergoing PD with ultrafiltration dysfunction exhibit elevated levels of VEGF in their peritoneal effluent. Furthermore, studies by Wang [59] and colleagues have shown that with increasing duration of dialysis, the levels of TGF-β1 and VEGF in serum and PDF rise, correlating with higher peritoneal transport rates and lower ultrafiltration volume. This suggests that PF and neovascularization can enhance peritoneal solute transport and reduce ultrafiltration volume. Moreover, studies have shown that elevated expression of HDAC1 increases the degradation of p53 through deacetylation, which can lead to a reduction in the repression of VEGF transcription and an increase in VEGF secretion [60]. Importantly, VEGF may also be involved in the generation of peritoneal neovascularization through various signaling pathways such as Wnt/β-catenin [61]. Therefore, inhibiting VEGF might improve pathological angiogenesis, thus providing a target for alleviating PF.

### Triggering of EMT

EMT is considered a triggering and initiating factor in the PF process and is a key link in the early occurrence and development of PF, primarily manifested by the loss of PMCs, fibrosis and thickening of the submesothelial compact area, angiogenesis, and hyaline vascular lesions [62,63]. However, the mechanisms underlying these effects remain unclear. During the EMT process, TGF-β serves as a key cytokine promoting its occurrence, and is an important factor in PF. Margetts et al. [64] established a rat peritoneal EMT model by introducing the *TGF-β* gene into the peritoneal cavity using an adenoviral vector. They found increased expression of genes associated with EMT and fibrosis, such as α-SMA, and the zinc finger regulator Snail. Within 4 to 7 days post-transfection, the PMCs layer exhibited positive expression of cytokeratin and α-SMA, and fibroblasts positive for cytokeratin and α-SMA were observed in the submesothelial tissue. These phenomena indicate that EMT likely occurs *in vivo* after TGF-β1 is overexpressed in the peritoneum. Research by Huang et al. [65] found that treatment of PMCs with TGF-β1 for 72 h resulted in significant upregulation of α-SMA protein expression and hydroxyproline secretion, while the expression of E-cadherin significantly decreased. Moreover, the cell morphology underwent noticeable changes, as the cells lost their regular cuboidal appearance and became elongated and spindle-shaped, leading to peritoneal mesothelial cell EMT. Therefore, TGF-β is one of the important promoting factors for the mesenchymal transformation of epithelial cells (including PMCs), capable of inducing the EMT process in human PMCs. Furthermore, reports indicate that HDAC inhibition can regulate fibrosis by reducing TGF-β-triggered myofibroblast differentiation and decreasing inflammatory cytokines [10].

## HDACs promote PD-related PF

In PF, HDAC acts as key regulatory factor in promoting the progression of fibrosis through various mechanisms such as activating pro-fibrotic signaling pathways, facilitating inflammatory responses, and inducing myofibroblast activation [11]. Based on different HDAC subtypes, we have summarized the specific roles of HDAC in promoting PF.

### Class I histone deacetylase

**Figure 2.**
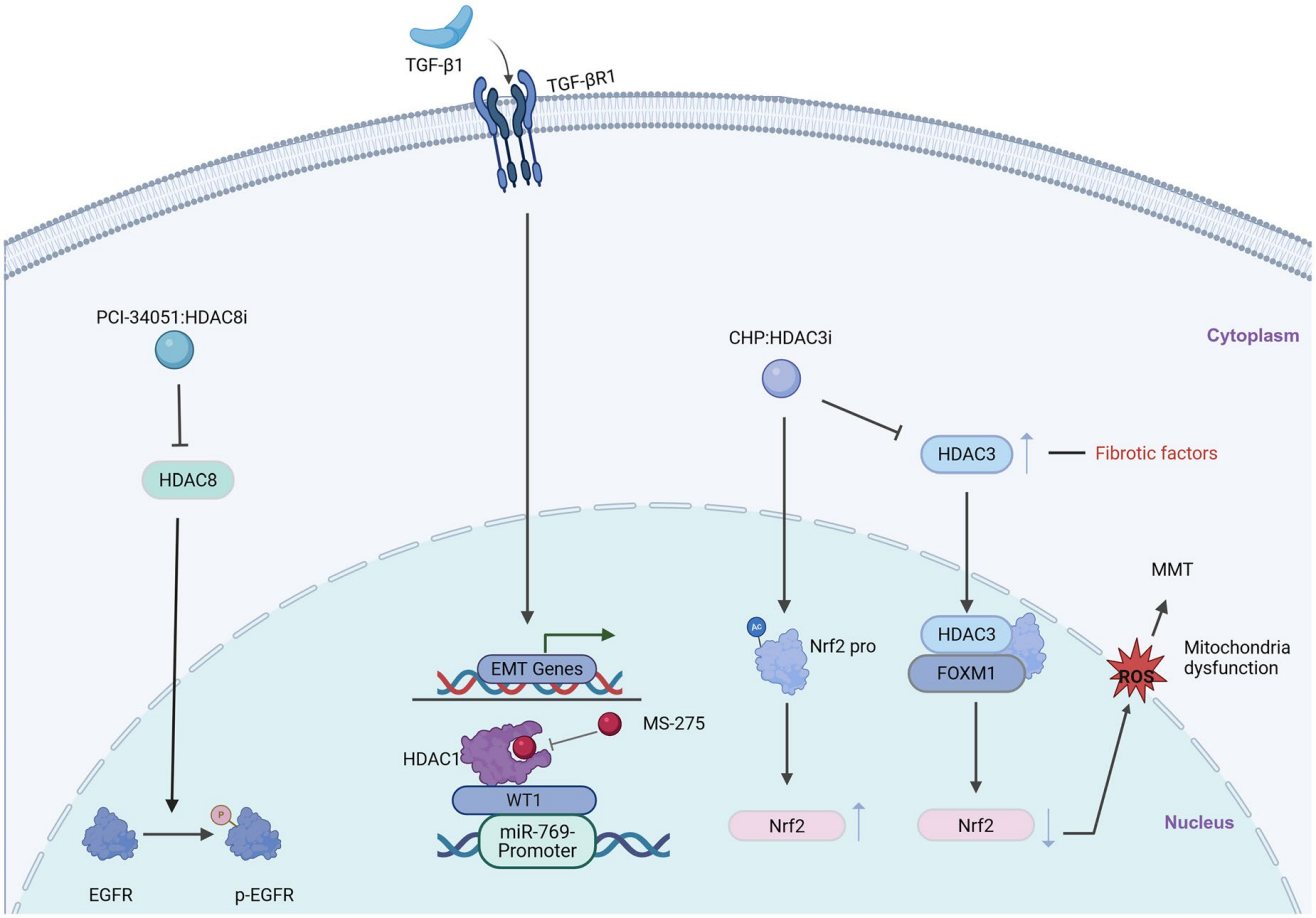
Some signaling pathways of Class I HDAC regulating PF during the injury phase. In this condition, the anti-fibrotic miR-769-5p is downregulated. HDAC1 inhibition may promote MMT reversal through WT1-induced miR-769-5p expression. Furthermore, CHP blocks HDAC3 activity, leading to an increase in the transcription of Nrf2 which reduces oxidative stress associated with progressive peritoneal injury. The specific selective inhibitor PCI-34051 of HDAC8 can block phosphorylation of the EGFR, reduce cell apoptosis, and inhibit EMT. TGF-β1, transforming growth factor beta receptor 1; HDAC8, histone deacetylase 8; EGFR, epidermal growth factor receptor; p-EGFR, Phosphorylated-EGFR; EMT, epithelial–mesenchymal transition; HDAC1, histone deacetylase 1; WT1, Wilms tumor protein; MS-275, Entinostat; HDAC3, histone deacetylase 3; FOXM1, Forkhead Box M1; Nrf2, Nuclear factor-erythroid factor 2-related factor 2; MMT, mesothelial-to-mesenchymal transition; ROS, reactive oxygen species.

Over the past few years, subtype-selective inhibitors of HDACs have been developed, allowing for the study of the functional roles of individual HDACs in PF. Recent research has identified three Class I HDAC subtypes - HDAC1, HDAC3, and HDAC8 - as potential therapeutic targets for PF (Figure 2).

- HDAC1

Bontempi et al. [66] conducted preclinical research experiments on effluent PMCs and the human mesothelial cell line Met-5A treated with TGF-β1 (2 ng/ml), and discovered that the expression of WT1 and its binding to the miR-769-5p promoter were both increased upon HDAC1 inhibition and diminished upon treatment with TGF-β1. Additionally, the ectopic expression of miR-769-5p was sufficient to promote EMT reversal, limiting the migration and invasion of PMCs, while silencing miR-769-5p further enhanced the expression of mesenchymal genes. This demonstrates that the HDAC1-WT1-mir-769-5p axis controls the epithelial-mesenchymal transitions in PMCs by influencing cell proliferation, and affects the occurrence of PF. Therefore, in the pathogenesis of PF, HDAC1 regulates the transcription of genes such as TGF-β, affects cell proliferation, promotes the initiation of fibrosis, and forms a fibrosis-promoting pathway mediated by HDAC1, which is initiated by TGF-β and involves EMT.

- HDAC3

Liu et al. [13] established an *in vitro* cell model under high glucose and LPS culture conditions. The experimental group pretreated HMrSV5 cells with 1,25 (OH)_2_D_3_ and found that exposure to high glucose and LPS resulted in inhibited cell proliferation and increased apoptosis, along with a trend of differentiation toward a fibroblast-like phenotype. When 1,25(OH)_2_D_3_ was present or absent, and Wnt agonist 1 (a Wnt signaling pathway activator) was applied, they found that 1,25(OH)_2_D_3_ could reverse this process by downregulating HDAC3 and upregulating the vitamin D receptor (VDR). Wnt agonist 1 increased the expression of EMT-related biomarkers, while 1,25(OH)_2_D_3_ inhibited their expression. Thus, in PMCs cells, HDAC3 inhibits the expression of the VDR gene. When the HDAC3 expression is inhibited, VDR expression is upregulated, thereby inhibiting EMT and ultimately slowing PF progression. In subsequent research, Kim et al. used human primary cultured peritoneal mesothelial cells (PMCs) and found that Cyclo(His-Pro) treatment dose-dependently improved the rTGF- β-induced phosphorylated HDAC3 and fibronectin expression, leading to increased Nrf2 transcription, reduced fibrosis (fibronectin, collagen 1 A) and inflammation (ICAM-1, pP65) markers, alleviated fibrosis damage, thereby preventing PMCs from developing fibrosis. In the 1990s, researchers extracted cDNA encoding HDAC3 from humans. Analysis of the amino acid sequence encoded by the HDAC3 open reading frame revealed approximately 50% homology with the HDAC1 and HDAC2. This suggests that the specific mechanisms of action of HDAC1-3 in PF may be similar [14,67].

- HDAC8

The impact of HDAC8 (another important subtype of the Class I HDAC family) on PF has also been studied. HDAC8 is highly expressed in a mouse model of PF induced by high-glucose PD solution. The specific and selective inhibitor of HDAC8, PCI-34051, can block EGFR phosphorylation and the activation of its downstream signaling pathways ERK1/2 and STAT3/HIF-1α, reduce cell apoptosis, inhibit EMT and M2 macrophage polarization, and alleviate the EMT process, thereby mitigating the progression of fibrosis [68].

Furthermore, studies [15] have shown that experiments conducted using PMCs isolated from the effluent of peritoneal dialysis patients revealed that MS-275 (a HDAC1-3 inhibitor) can inhibit the phosphorylation of Smad3, EGFR and STAT3, thereby promoting the downregulation of mesenchymal markers (MMP2, PAI-1, TGF-β) and the upregulation of epithelial markers (E-cadherin), suppressing the EMT of PMCs, restoring the epithelial-like morphology, and effectively inhibiting the fibrotic ability of PMCs.

The above data indicate that HDAC1 affects cell proliferation by regulating the transcription of genes downstream of the TGF-β signaling pathway (miR-769-5p, WT1 transcription factor), thereby promoting fibrosis; HDAC3 regulates the expression of genes related to Wnt/β-Catenin and Nrf2 signaling pathways, controlling the cell cycle and peritoneal inflammatory response, thereby contributing to PF; HDAC8, although not clearly related to the TGF-β signaling pathway, directly acts on the EGFR/ERK1/2 signaling pathway in PMCs, induces M2 macrophage polarization, and triggers peritoneal inflammation to promote PF.

Meanwhile, HDAC1 and HDAC2 can form a complex called Sin3, which cooperatively performs deacetylation functions and participates in cell cycle regulation and the initiation of fibrosis (affecting cell cycle inhibitory genes like p21 and fibrosis-related genes like TGF-β). Additionally, HDAC1/2 enhance NF-κB activity by inhibiting NF-κB inhibitory factors (such as IκBα), promoting the release of inflammatory factors (TNF-α, IL-6); HDAC3 enhances the activity of inflammation-related enzymes in macrophages through deacetylation modification, and cooperatively amplifies inflammation [69,70].

In conclusion, although HDAC1, 2, 3, and 8 all belong to the same class, due to the relatively low homology between HDAC8 and HDAC1, 2, and 3, their functions are relatively independent. HDAC8 mainly promotes disease progression by influencing the inflammatory response, while HDAC1 and 3 mainly participates in the PF process through cell proliferation and cell cycle regulation.

### Class IIa histone deacetylase

**Figure 3.**
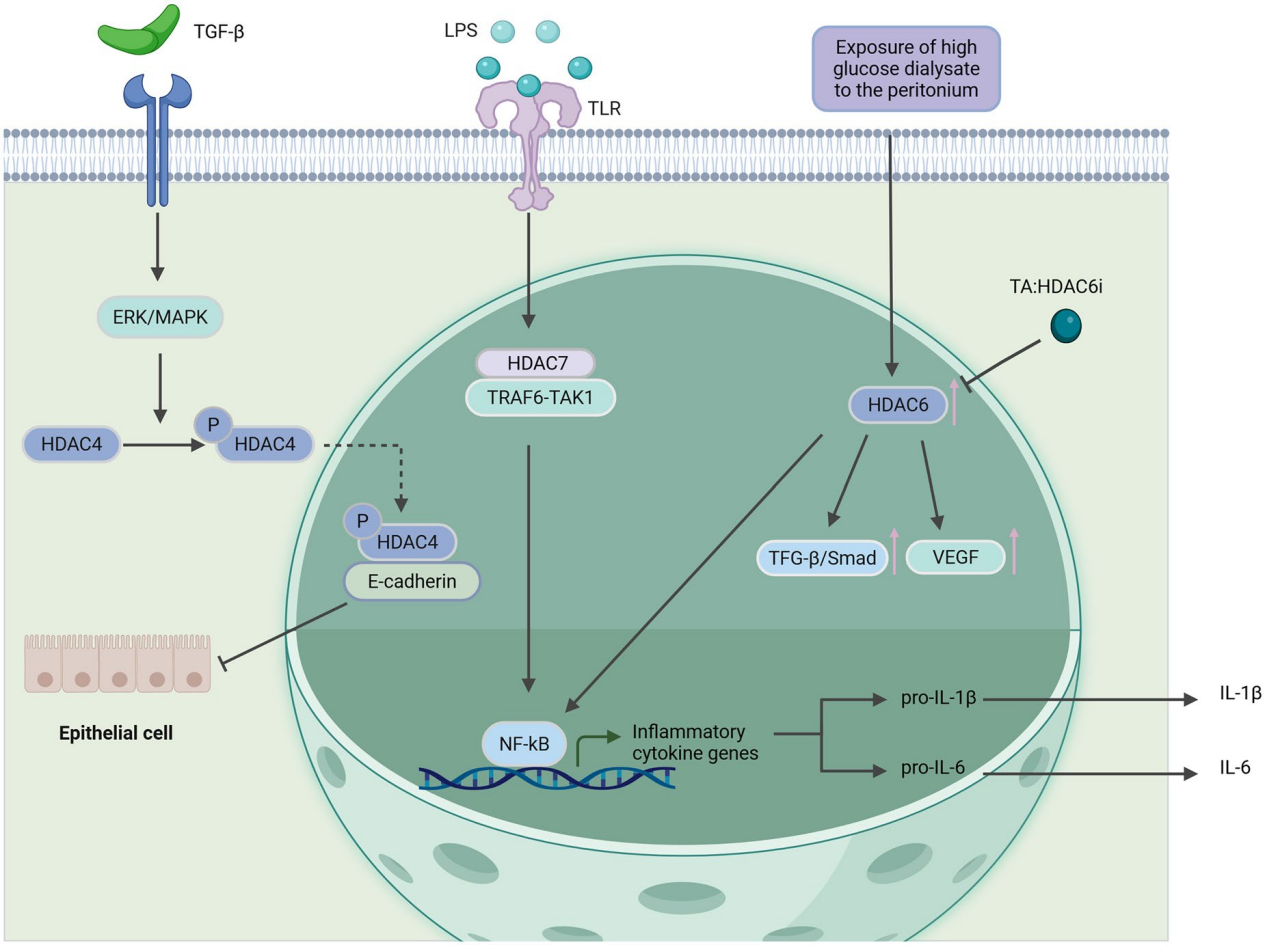
Some signaling pathways of Class II HDAC regulating PF during the injury phase. TGF-β1 stimulation can promote the phosphorylation and nuclear translocation of HDAC4 by activating the ERK/MAPK pathway. It inhibits histone acetylation by directly binding to the E-cadherin promoter region, thereby directly blocking the maintenance of the mesothelial phenotype. Under LPS stimulation, HDAC7 binds to the TRAF6-TAK1 complex in cells through a non-enzymatic mechanism, activating the NF-κB pathway to enhance the expression of pro-inflammatory target genes such as *IL-1β* and *IL-6*. Furthermore, exposure to high-glucose dialysate upregulates HDAC6 in the peritoneum, subsequently inducing a pro-inflammatory response (the activation of NF-κB) and triggering angiogenesis by increasing VEGF production, and the activation of the TGF-β/Smad pathway. All of these reactions are inhibited by TA, a highly selective HDAC6 inhibitor. TGF-β, transforming growth factor beta receptor; ERK, extracellular regulating kinase; mitogen-activated protein kinase; HDAC, histone deacetylase; p, phosphorylation; LPS, lipopolysaccharide; TLR, Toll like receptors; TRAF, tumor necrosis factor receptor-associated factor; TAK1, Transforming Growth Factor-β-Activated Kinase 1; NF-κB, Nuclear factor kappa B; Smad, drosophila mothers against decapentaplegic protein; VEGF, vascular endothelial growth factor; IL-1β, Interleukin-1β; IL-6, Interleukin-6; TA, Tubastatin A.

Currently, there are relatively few studies on Class IIa HDACs in peritoneal fibrosis. Class IIa HDACs can dynamically shuttle between the nucleus and cytoplasm through a phosphorylation-dephosphorylation cycle, influencing the transcription of fibrosis-related genes (Figure 3).

- HDAC4

Currently, there is no research directly indicating that HDAC4 plays a role in PF. However, studies have shown that in preclinical experiments of mouse renal fibrosis induced by unilateral ureteral obstruction (UUO), the expression of HDAC4 and α-SMA in renal tissues is activated, and the expression of the pro-fibrotic factor TGF-β1 is also significantly elevated. Moreover, after continuous intraperitoneal injection of 25 mg/kg/day tasquinimod (a specific HDAC4 inhibitor) for 6 days post-surgery, and in mice with genetically deleted HDAC4 in renal tubular cells, it was found that inhibition of HDAC4 could reduce G2/M cell cycle arrest in renal tubular epithelial cells, decrease the expression of TGF-β1, and suppress the activation of the pro-fibrotic signaling pathways (Smad3, and STAT3), while also preserving the expression of Klotho (a renal protective protein) at the transcriptional level [16]. It can be seen that HDAC4 primarily promotes fibrosis progression by regulating pro-fibrotic pathways.

- HDAC5

In the development of PF, HDAC5 plays a role in regulating inflammatory responses. Studies have shown that in the resting state (normal peritoneal tissue), HDAC5 inhibits NF-κB activity through deacetylation and reduces the release of inflammatory factors. However, under high glucose stimulation (intraperitoneal injection of 10 mL/kg/day of 4.25% PDF, continuously for 28 days), the phosphorylation level of HDAC5 decreases, and its nuclear retention time is prolonged, which promotes the activation of the TGF-β1/Smad3 pathway and accelerates fibrosis [17,71].

- HDAC7

Although there is no direct research proving that HDAC7 plays a role in PF, existing studies have shown that in LPS-stimulated macrophages and in mouse models, HDAC7 binds to the TRAF6-TAK1 complex through a non-enzymatic mechanism, activating the MAPK/NF-κB pathway, promoting the secretion of pro-inflammatory factors such as IL-6 and TNF-α, and providing an inflammatory microenvironment for fibrosis [72]. HDAC7 integrates inflammatory and fibrotic signals by regulating pathways such as NF-κB and STAT3, and participates in the development of fibrotic diseases [73].

- HDAC9

Currently, there have been no reports on HDAC9 research in PF. However, in renal fibrosis, HDAC9 inhibits STAT1 acetylation, reduces its nuclear translocation and the expression of downstream fibrotic genes (such as CTGF), causing renal tubular epithelial cell cycle arrest in the G2/M phase, promoting cell senescence and the release of fibrotic factors [74,75]. In the progression of fibrotic diseases, HDAC9 is a key factor in cell cycle regulation.

In summary, Class IIa HDACs mainly affect transcriptional regulation through nuclear-cytoplasmic shuttling. Specifically, HDAC4 regulates and influences the fibrosis process through pro-fibrotic pathways, while HDAC5 stimulates the release of inflammatory factors to promote PF progression. However, there are few reports on HDAC7, and HDAC9 research in PF. The HDAC4/7 complex can promote the secretion of inflammatory factors and further activate the TGF-β/Smad pathway, enhancing HDAC4 expression, thereby forming an “inflammation - fibrosis” vicious cycle; while HDAC9 can inhibit the activity of STAT1 and STAT3, blocking pro-fibrotic signal transduction, and forming a counterbalance with HDAC4, and HDAC7.

### Class IIb histone deacetylase

- HDAC6

Recent studies have emphasized that HDAC6 is involved in the development of PF and is significantly overexpressed in fibrotic peritoneal tissues, which is mainly related to pro-fibrotic (TGF-β1) and pro-inflammatory mediators (IL-6) [19,20,76].

Notably, IL-6 upregulates HDAC6 expression, thereby linking inflammation and fibrosis [19]. HDAC6 promotes the progression of fibrosis by regulating the TGF-β/Smad3 pathway, which regulates fibroblast activation and ECM deposition in multiple tissues (including the peritoneum) [20].

Importantly, HDAC6 inhibition has shown promise in alleviating PF [18,77]. Xu et al. in a PDF-induced PF mouse model, administered the selective HDAC6 inhibitor tubastatin A (TA) to inhibit TGF-β1-induced EMT in PMCs, reduce the expression of collagen I and α-SMA, and prevent fibrosis in the mouse model. These effects are mediated by regulating key signaling pathways (including the NF-κB inflammatory signaling pathway) [19]. Additionally, HDAC6 inhibition reduces angiogenesis by downregulating VEGF and inhibiting Wnt/β-catenin signaling [20].

HDAC6 is also associated with the inflammatory response during PF. Blocking HDAC6 reduces macrophage recruitment and their polarization toward the pro-fibrotic M2 phenotype by disrupting the TGF-β/Smad and IL-4/STAT6 pathways [76]. Moreover, it weakens IL-6-driven ECM deposition by simultaneously regulating the JAK2/STAT3 and TGF-β/Smad3 pathways, demonstrating its central role in mediating the crosstalk between inflammation and fibrosis [78].

Apart from its well-known role as a histone modifier, HDAC6 interacts with multiple non-histone substrates, including α-tubulin, heat-shock protein 90 (HSP90), peroxiredoxins, and TGF-β [79–81]. However, it remains unclear how TGF-β activates HDAC6. The deacetylation of non-histone proteins such as α-tubulin by HDAC6 may provide a structural basis for EMT. Studies have shown that HDAC6 specifically deacetylates α-tubulin at the Lys-40 position, stabilizing microtubules, influencing cell movement and promoting ECM hardening, thereby leading to organ fibrosis [82]. Although these findings highlight the potential of HDAC6 inhibitors as therapeutic agents for PF, the underlying mechanisms remain unclear. Future studies should aim to elucidate the precise molecular interactions controlled by HDAC6, evaluate the clinical applicability of highly selective HDAC6 inhibitors when combined with existing anti-fibrotic and anti-inflammatory targets.

- HDAC10

To date, the other subtype of Class IIb HDAC, HDAC10, has not been reported to be elevated in PF. Moreover, there are no specific inhibitors for HDAC10, and the exact mechanism of its action in PF remains unclear.

### Class III histone deacetylase

**Figure 4.**
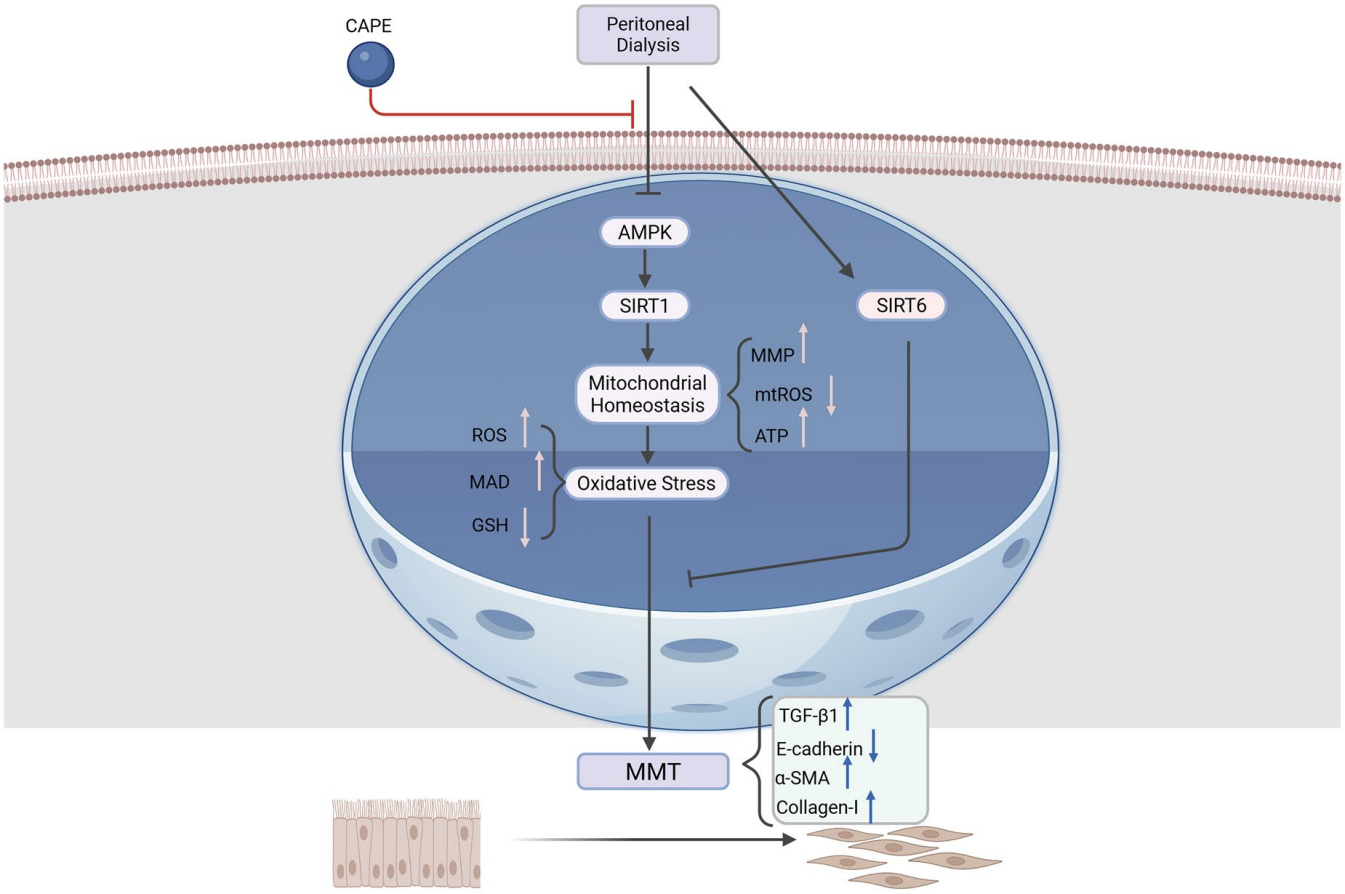
The molecular mechanism by which SIRT1 inhibits PF during the injury phase. Under PD conditions, the AMPK/SIRT1 pathway is downregulated, disrupting mitochondrial homeostasis, which in turn leads to oxidative stress. This elevated oxidative stress causes EMT, ultimately resulting in PF. Interestingly, all these responses can be inhibited by CAPE, thereby alleviating PD-induced EMT and PF. CAPE, caffeic acid phenethyl ester; AMPK, Adenosine 5′-monophosphate (AMP)-activated protein kinase; SIRT1, Silent mating type information regulation 2 homolog-1; MMP, mitochondrial membrane potential; mtROS, Mitochondrial reactive oxygen species; ATP, Adenosine triphosphate; ROS, reactive oxygen species; MDA, Malondialdehyde; GSH, glutathione; MMT, mesothelial-to-mesenchymal transition; TGF-β1, transforming growth factor beta receptor 1; α-SMA, α-Smooth muscle actin.

The known members of the SIRTs currently include SIRT1 to SIRT7. SIRTs are highly conserved and play significant roles in various biological processes such as cellular metabolism, aging, and stress responses (Table 2). It is currently believed that members of the Class III HDAC family, particularly SIRT1 and SIRT6, are primarily associated with the occurrence of PF (Figure 4).

**Table 2.** Functional differences of HDAC Class III.

Category	Mechanism of Action	Cell localization	Signal pathway	Fibrosis-related effects	References
SIRT1	Mitigate inflammatory response.	Nucleus/Cytoplasm	AMPK/SIRT1, TGF - β/Smad	Alleviate inflammation	Guo et al. (2021) [21]
SIRT2	Influence cell migration and inflammatory response.	Cytoplasm	Microtubule stabilization, TGF-β/Smad	Inhibition of migration and activation.	He et al. (2018) [83]
SIRT3	Regulating mitochondrial energy metabolism and the inflammatory response.	Mitochondria	SOD2/ROS, AMPK/PGC-1α	Improvement of mitochondria and antioxidant mechanisms.	Zhang et al. (2019) [12]
SIRT4	Regulate amino acid metabolism and energy metabolism.	Mitochondria	Mitochondrial metabolism, TGF-β	Metabolic regulation	Mazumder et al. (2020) [84]
SIRT5	Influence on metabolism	Mitochondria	Urea cycle, fatty acid oxidation	Metabolic regulation	
SIRT6	Regulation of DNA repair and metabolic balance	Nucleus	NF-κB, TGF-β, Telomere Regulation	Inhibit the TGF-β/NF-κB pathway; maintain ECM balance.	Shi et al. (2024) [24]
SIRT7	Participate in cell proliferation.	Nucleus	rRNA Transcription, TGF-β/Smad	Regulation of rRNA transcription affects cell proliferation.	Chen et al. (2017) [85]

Abbreviations: SIRT, silent mating type information regulation 2 homolog; AMPK, adenosine 5′-monophosphate (AMP)- activated protein kinase; TGF-β, transforming growth factor-β; Smad, drosophila mothers against decapentaplegic protein; SOD2: superoxide dismutase 2; ROS, reactive oxygen species; PGC-1α: peroxisome proliIerators-activated receptor γ coactivator lalpha; NF-κB, nuclear factor kappa B; ECM: extracellular matrix; rRNA: ribosomal RNA.

- SIRT1

SIRT1 is a widely studied subtype of class III HDACs. SIRT1 is primarily located in the cell nucleus, and its activation relies on NAD+ as a coenzyme; resveratrol is its activator. Research by Guo et al. [21] found that the expression of SIRT1 decreases in the peritoneum following PD. They established an animal model using SIRT1^-/-^ mice and discovered that SIRT1 knockout exacerbates PF both *in vivo* and *in vitro*. Furthermore, SIRT1 upregulation effectively improves PF by inhibiting the secretion of extracellular matrix proteins induced by TGF-β signaling. This suggests that SIRT1 has an anti-fibrotic effect. Lu et al. [22] treated PMCs with PDF, and the results showed inhibition of the AMPK/SIRT1 pathway, depolarization of the mitochondrial membrane potential, excessive production of mitochondrial reactive oxygen species (ROS), reduced ATP synthesis, and induction of mesothelial-to-mesenchymal transition (MMT). When caffeic acid phenethyl ester (CAPE) was added to the PDF, CAPE activated the AMPK/SIRT1 pathway, thereby inhibiting mitochondrial membrane potential depolarization, reducing mitochondrial ROS production, and maintaining ATP synthesis. Based on these data, targeting SIRT1 to modulate the AMPK/SIRT1 and TGF-β/Smad pathways may inhibit inflammatory responses and alleviate oxidative stress damage, potentially reducing PF progression.

- SIRT6

Shi et al. [24] observed changes in SIRT6 levels and MMT after PDF intervention using HMrSV5 cells. They found that SIRT6 levels in PD effluents (PDEs) were negatively correlated with the duration of PD, total glucose exposure, TGF-β1 and IL-6 levels, and the ratio of creatinine in dialysis fluid to plasma (Cr4hD/P), while positively correlated with ultrafiltration. SIRT6 overexpression resulted in increased E-cadherin expression in PMCs, along with decreased expression of vimentin and TGF-β1. This indicates that SIRT6 expression can inhibit MMT in PMCs, alleviating PD-related PF. Furthermore, SIRT6 influences cellular energy metabolism by regulating glycolysis and lipid metabolism (such as inhibiting HIF-1α stability), reducing oxidative stress damage induced by high-glucose dialysis fluid [86]. Therefore, SIRT6 has a protective effect on the peritoneum.

- SIRT2, 3, 4, 5, 7

SIRT2 is also localized in the nucleus and is significantly elevated in patients with liver fibrosis, cardiac fibrosis, and renal fibrosis. However, there has been relatively little research on the specific regulatory mechanisms of SIRT2 in PF. Current studies suggest that it may mediate the PF progression through TGF-β signaling pathways. TGF-β1 induces SIRT2 upregulation in fibroblasts, which in turn promotes the activation of myofibroblasts as well as the deposition of α-SMA, collagen III, fibronectin, and other ECM components. This process can be significantly inhibited by AGK2, a SIRT2-specific inhibitor [83]. However, no effect of SIRT2 on epithelial to mesenchymal transition in cells was observed.

SIRT3 is mainly located in the mitochondrial matrix and serves as the primary regulatory factor for mitochondrial deacetylation. Moreover, reports indicate that SIRT1 and SIRT3 can exert protective effects against renal fibrosis by inhibiting inflammatory responses and cell apoptosis, as well as regulating energy metabolism [87].

The regulatory mechanism of SIRT4 in PF has not yet been fully determined. SIRT4 is a deacetylase associated with cellular stress responses. In metabolic reprogramming, it regulates various factors such as NAD+ and AMP-activated protein kinase (AMPK), and exerts antioxidant protection and repair effects. It can also regulate the myofibroblast transdifferentiation and subsequent collagen fiber deposition by modulating glutamine (Gln) metabolism [23,84]. SIRT4 primarily regulates intracellular amino acid metabolism and energy metabolism.

SIRT5 is also a mitochondrially localized deacetylase, yet there are few published studies regarding its regulatory mechanisms in CKD and PF. It primarily participates in metabolic regulation, such as fatty acid oxidation and glutamine metabolism [88]. It may affect energy metabolism disorders related to fibrosis in patients undergoing peritoneal dialysis.

SIRT7 has been shown to inhibit the TGF-β1/Smad signaling pathway by deacetylating Smad4 and reducing Smad3 levels, thereby regulating fibrosis [85].

In summary, there is currently limited understanding of the role of Class III HDACs in PF. SIRT1 primarily alleviates the inflammatory response by modulating the AMPK/SIRT1 and TGF-β/Smad signaling pathways, thereby suppressing PF. In contrast, SIRT6 primarily inhibits the transcription of fibrosis-associated genes directly.

### Class IV histone deacetylase

HDAC11 is the sole isoform of Class IV HDAC. In PF, there has been limited research focusing on HDAC11. However, studies have shown that in a mouse kidney fibrosis model, HDAC11 expression significantly increased. Additionally, in cultured renal tubular epithelial cells, treatment with angiotensin II (Ang II) also led to HDAC11 upregulation. In further research, HDAC11 inhibits the transcription of Kruppel-like factor 15 (KLF15) by interacting with activator protein 2 (AP-2α), thereby promoting kidney fibrosis [25]. Thus, HDAC11 participates in the progression of fibrotic diseases by regulating the transcription factor KLF15.

## Summary and outlook

- Mechanism of Action and Clinical Significance

In this review, we summarize recent research findings on the role of HDACs in PF. The core cause of PF is the long-term peritoneal exposure to “injury stimuli”, where EMT is the core cellular basis of fibrosis, the inflammatory response is the “amplifier” of fibrosis, and ECM metabolic imbalance is the ultimate manifestation of fibrosis. Each component is interrelated through TGF-β1, NF-κB, Smad and other signaling pathways, forming an irreversible vicious cycle. Therefore, therapeutic intervention needs to target multiple pathways (such as inhibiting TGF-β1 and regulating inflammation) to delay or block the fibrosis progression. As important enzymes in the histone modification process, HDACs can exacerbate the inflammatory response by regulating the inflammatory factor expression and immune cell function, enhance the crosstalk of the TGF-β signaling pathway, and participate in multicellular interactions during PF through multiple mechanisms. HDAC1 and HDAC3 affect ECM metabolism, while HDAC4 and SIRT6 control EMT and fibroblast activation. However, all HDAC types are related to the inflammatory response. HDAC5, HDAC6, HDAC8 and SIRT1 act as intermediary factors that accelerate cell phenotype transformation and ECM deposition through immune cell infiltration or cytokine release, ultimately forming an “inflammation-fibrosis” vicious cycle. This suggests that different HDAC types have overlapping functions despite clear classification. Therefore, studying functionally similar HDACs together represents a valuable research approach. However, due to differences in enzyme structure, cofactor requirements, and substrate specificity among different HDAC subtypes, their targeted drugs (inhibitors/activators) exhibit distinctly different characteristics, resulting in significant variations in drug tolerance, selectivity, and toxicity profiles. This also indirectly indicates that they have marked differences in terms of drug tolerance, selectivity, and toxicity profiles.

Importantly, current clinical interventions for PD (biocompatible PDFs, glucose-sparing regimens, icodextrin, ACEi/ARB, SGLT2 inhibitors in early CKD, and peritonitis prevention bundles) directly target mechanisms of PD membrane damage (inflammation, fibrosis, angiogenesis) or indirectly improve the peritoneal microenvironment to protect the PD membrane, yet they still have clear limitations. PDF are costly and difficult to implement widely at the primary care level; glucose-sparing regimens require precise adjustment, and some patients may experience ultrafiltration insufficiency; icodextrin carries a rare risk of allergic reactions and long-term use may increase the likelihood of peritoneal adhesions; ACEi/ARB can cause hypotension or hyperkalemia in certain patients; the use of SGLT2 inhibitors in early CKD is limited by eGFR levels (contraindicated in patients with excessively preserved renal function) and may increase the risk of urinary tract infections; peritonitis prevention bundles are highly dependent on patient adherence and are less effective against complex infections such as fungal infections. Therefore, gaining an in-depth understanding of HDAC mechanisms in PF holds promise as a novel therapeutic strategy for fibrotic diseases.

- Limitations

HDACi show clinical potential in the treatment of PF. However, clinical data indicate that the use of HDACi is often accompanied by complications: gastrointestinal reactions caused by vorinostat (a broad-spectrum HDACi) are common and prominent, and hematological toxicity caused by chidamide (a Class I HDACi) requires close monitoring. This is due to the combination of the unique physiological and pathological characteristics of this population (renal function impairment, multiple comorbidities, dialysis dependence) with the pharmacological properties of broad-spectrum HDAC inhibitors (non-selective inhibition, widespread systemic effects), leading to an increased risk of adverse reactions. HDACi mainly consist of three pharmacophores: the cap group (such as the hydroxamic acid group), the linker group, and the zinc-binding group (ZBG, the functional group). The structure of the pharmacophore determines the therapeutic effect of HDACi. Currently marketed HDACi mainly target Class I and Class II HDACs, specifically HDAC1, 2, 3, and 8. This corresponds to the treatment targets studies in PF research. Regarding side effects, vorinostat broadly inhibits Class I and IIb HDAC and has strong chelating ability, making it unsuitable for the treatment of PF due to its potential to cause proteinuria. Chidamide contains a benzamide group, has high selectivity for Class I HDACi and causes relatively mild side effects (lower hematological toxicity incidence than vorinostat). Despite good therapeutic effects, HDACi selection for PF treatment must be based on specific clinical presentations. Overall, Class I HDACi have greater prospects for PF treatment. Therefore, future research must balance HDACi efficacy and toxicity, address side effects while maintaining therapeutic efficacy. In subsequent clinical studies, it is recommended to conduct small-sample, dose-escalation Phase I/II clinical trials to evaluate the ‘dose-toxicity’ relationship in patients with different stages of peritoneal function. Particular attention should be paid to monitoring myelosuppression, infections, metabolic abnormalities, and QTc interval, and to establishing a dose-adjustment formula based on eGFR. And focus on developing HDACs with higher specificity and selectivity, while screening HDACi with high specificity and low toxicity.

In animal model studies, intraperitoneal injection of HDACi can inhibit PF progression in mice. First, based on previous literature reports, most mouse models of PF were constructed by intraperitoneal injection of PDF or chlorhexidine gluconate (CG), which simplifies the PF formation process. However, clinical PF results from PD complications, creating differences between these models and clinical reality. Constructing PF models by performing 5/6 nephrectomy in mice followed by PD treatment may be more clinically relevant. Second, intraperitoneal injection allows more precise peritoneal targeting, while the currently marketed HDACi are mainly taken orally, making precise treatment difficult. Additionally, HDACi are widely used for diffuse tumor treatment, indicating broad therapeutic effects but also highlighting the lack of specificity of current HDACi. Current research focuses primarily on exploring mechanisms of new HDAC targets and evaluating therapeutic effect of HDACi. Few studies examine other organ function (physiological and biochemical) during PF treatment in rodents, so future research must pay more attention to toxic side effects while achieving therapeutic goals.

- Future Direction

Current research on PD-related PF remains insufficient. PF is a dynamic process with complex multicellular properties; therefore, it is necessary to construct reproducible models through methods such as single-cell RNA sequencing to clarify process related changes and identify epithelial-mesenchymal heterogeneity during cellular transformation. Additionally, our understanding of histone acetylation modification in PF is incomplete. Current approaches are simplistic, focusing only on phenotypes and pathway research, with insufficient mechanistic clarity. For Class I HDACs, molecular docking methods can help clarify target site differences among non-homologous sequences and further elucidate their roles through peritoneal-specific gene knockout mice. For Class IIa HDACs, which have been rarely studied, protein expression patterns in clinical samples should be established first. For HDAC6, three-dimensional mesothelial cell culture can better simulate the human microenvironment for more clinically relevant research. For Class IV HDACs and Class III HDACs, single-cell and multi-omics data can better clarify their properties. More importantly, HDACi research requires “precisely chelation, target binding, and reduced toxicity” [89].

In conclusion, HDACs play a key role in mediating PF progression. HDACi are potential therapeutic agents for PF. Targeting HDACs represents a promising treatment strategy for PF.

## Data Availability

Data sharing is not applicable to this article as no new data were created or analyzed in this study.
